# Feasibility of Artificial Intelligence-Processed Low-Dose Cone-Beam Computed Tomography in Dental Imaging

**DOI:** 10.3390/bioengineering13030304

**Published:** 2026-03-05

**Authors:** Tae-Yoon Park, Seung-Eun Lee, Sang-Yoon Park, Sung-Woon On, Sang-Min Yi, Byoung-Eun Yang, Soo-Hwan Byun

**Affiliations:** 1 Department of Oral and Maxillofacial Surgery, Hallym University Sacred Heart Hospital, Anyang 14066, Republic of Korea; 2 Institute of Clinical Dentistry, Hallym University, Chuncheon 24252, Republic of Korea; 3 Dental Artificial Intelligence and Robotics R&D Center, Hallym University Medical Center, Anyang 14066, Republic of Korea; 4 Division of Oral and Maxillofacial Surgery, Hallym University Dongtan Sacred Heart Hospital, Hwaseong 18450, Republic of Korea

**Keywords:** artificial intelligence, cone-beam computed tomography, low-dose imaging, radiation exposure, image quality enhancement, pediatric dentistry

## Abstract

Cone-beam computed tomography (CBCT) plays an important role in dental diagnosis; however, radiation exposure remains a concern. This study evaluated the feasibility of artificial intelligence (AI)-based image processing for improving image quality in low-dose CBCT. CBCT scans were acquired from a single healthy adult male at three radiation dose levels (10%, 20%, and 100% of the standard dose), and each dataset was subsequently processed using an AI-based image enhancement model. Five dental specialists independently assessed image quality using a 6-point scoring system across 12 anatomical and diagnostic criteria, including anatomical visibility, structural delineation, and overall diagnostic acceptability. The AI-processed 20% dose images showed no statistically significant difference in image quality compared with the 100% raw dose images (median 4.45, range 3.50–5.30 vs. median 5.05, range 4.50–5.50; *p* > 0.05). In contrast, the AI-processed 10% dose images demonstrated significantly lower scores (*p* = 0.0074), and the AI-processed 100% dose images were rated lower than the corresponding raw images. These preliminary findings suggest that AI-assisted enhancement may partially mitigate image quality degradation associated with moderate CBCT dose reduction. Further large-scale studies involving diverse patient populations and clinical settings are required to validate these results.

## 1. Introduction

Cone-beam computed tomography (CBCT) is essential in dentistry, offering three-dimensional visualization of anatomical landmarks, supporting diagnosis, treatment planning, and surgical guidance across various dental procedures [1,2,3,4,5]. However, radiation risk remains a significant concern, particularly for pediatric patients with higher radiosensitivity [6,7,8]. Low-dose CBCT protocols have been developed through adjustments to acquisition parameters; however, dose reduction often compromises image quality by increasing image noise and reducing spatial resolution, thereby limiting diagnostic utility [9,10,11,12,13,14,15].

Recent advancements in artificial intelligence (AI), particularly deep learning-based algorithms, have offered innovative solutions for mitigating image degradation in low-dose CT, significantly enhancing the image quality [16]. AI-based CBCT image enhancement can be broadly categorized into several approaches.

Convolutional neural networks (CNNs) are widely used in medical image analysis and have demonstrated potential in low-dose CBCT applications, including adaptive radiotherapy and mandibular segmentation, even in the presence of metal artifacts [17,18]. However, CNN-based approaches often require large labeled datasets and exhibit limited interpretability due to their black-box nature [19,20].

Generative adversarial networks (GANs) have also been applied to low-dose CT and CBCT image enhancement, enabling effective noise reduction while preserving anatomical details [21,22]. Nevertheless, GANs are highly dependent on training data quality and may introduce artifacts when inadequately trained, limiting their clinical reliability [22,23,24].

More recently, transformer-based models have been introduced for CT image denoising by leveraging self-attention mechanisms to capture long-range spatial dependencies; however, limitations related to interpretability and clinical transparency persist [22]. Attentional U-Net, a modified U-Net architecture that incorporates attention mechanisms, has demonstrated effectiveness in medical image enhancement by selectively focusing on relevant regions of interest without requiring external localization modules, thereby improving computational efficiency and diagnostic performance [25].

Although AI models have demonstrated improvements in specific maxillofacial applications, such as discrimination of maxillary sinus pathologies and mandibular third molars, their application to broad low-dose CBCT imaging, particularly under substantially reduced dose conditions, remains limited [26,27]. Previous research has primarily focused on a single tooth, demonstrating improved image quality through denoising and sharpness enhancement without compromising diagnostic accuracy [28].

Given the increased noise and reduced contrast in low-dose CBCT images, particularly affecting fine anatomical structures, an architecture capable of selectively emphasizing diagnostically relevant regions was required to extend AI applications beyond isolated teeth. Therefore, the Attention U-Net was selected for this study to adaptively focus on salient anatomical features while suppressing background noise.

In this study, we aimed to evaluate the feasibility of AI-processed low-dose CBCT images in comparison with standard-dose (100%) CBCT images. To minimize anatomical variability and enable an exploratory comparison of dose-dependent changes in image quality, this study employed a single-subject design. Specifically, this study investigated whether AI-based image enhancement can compensate for radiation dose reductions of 10% and 20% while maintaining clinically acceptable image quality.

## 2. Materials and Methods

### 2.1. Ethics Approval and Patient Consent

**Figure 1:**
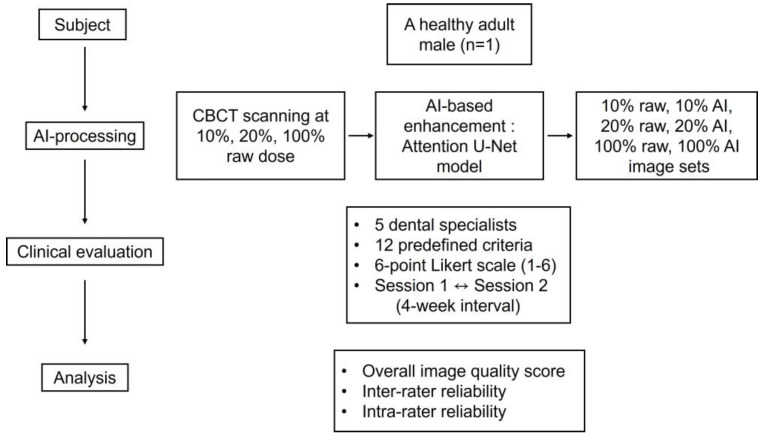
Workflow of the study design and evaluation process.

This study was conducted in accordance with the Declaration of Helsinki and approved by the Institutional Review Board of Hallym University Sacred Heart Hospital (IRB approval No. 2021-07-016, 14 September 2021). All CBCT examinations were performed solely for research purposes and were not based on a clinical indication. Written informed consent was obtained from the participant prior to imaging after a full explanation of the study protocol and potential radiation exposure (Figure 1).

### 2.2. CBCT Scanning Protocol

A single-subject, intra-individual study design was employed to enable direct comparison of CBCT images acquired under different radiation dose conditions while minimizing anatomical variability. The use of a single healthy adult male without maxillofacial pathology provided a consistent anatomical reference, allowing controlled assessment of technical image quality changes associated with dose reduction and AI-based post-processing. This approach was selected for this exploratory feasibility study to support consistent visual comparisons across dose levels while adhering to the ALARA (As Low As Reasonably Achievable) principle by limiting unnecessary radiation exposure. The limitations inherent to a single-subject design are acknowledged and are addressed in the Discussion. Scans were performed at three radiation dose levels: 10%, 20%, and 100% of the standard clinical dose. These initial scans, referred to as “raw images,” were not subjected to AI processing. Each raw image was subsequently processed using an AI-based image enhancement algorithm, resulting in three additional image sets termed “AI-processed images.” Six image sets were thus prepared and categorized according to the radiation dose level and AI processing status as follows: Image 1 (10% dose, raw image), Image 2 (10% dose, AI-processed), Image 3 (20% dose, raw image), Image 4 (20% dose, AI-processed), Image 5 (100% dose, raw image), and Image 6 (100% dose, AI-processed). CBCT was performed using a Bright CT scanner (Dentium; Suwon, Republic of Korea).

### 2.3. Dose–Area Product Measurement

Six CBCT image sets were labeled according to the radiation dose level (10%, 20%, or 100%) and AI processing status. Each image set exhibited a distinct dose–area product (DAP) value owing to variations in tube voltage (kVp), tube current (mA), exposure time (ms/projection), and number of projections. The field of view (FOV) was kept constant across all image sets.

The 100% dose raw image (Image 5) was acquired using the following scanning parameters: 95 kVp tube voltage, 11 mA tube current, FOV of 12 × 9.5 cm^2^, and 16 ms exposure time, resulting in a DAP of 1933.83 µGy·m^2^. To obtain 10% and 20% dose levels, the scanning parameters were adjusted accordingly.

**Figure 2:**
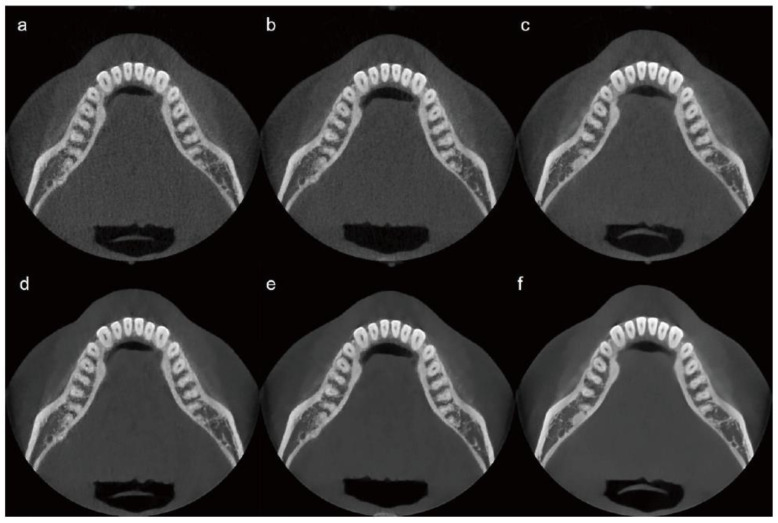
CBCT image sets categorized by radiation dose level and AI processing status. (**a**) 10% dose, raw image; (**b**) 20% dose, raw image; (**c**) 100% dose, raw image; (**d**) 10% dose, AI-processed image; (**e**) 20% dose, AI-processed image; (**f**) 100% dose, AI-processed image. AI, artificial intelligence; CBCT, cone-beam computed tomography.

For the 10% dose image set, the parameters were 85 kVp, 8.5 mA, and 16 ms, resulting in a DAP of 193.38 µGy·m^2^. For the 20% dose image set, the parameters were adjusted to 85 kVp, 9 mA, and 16 ms, yielding a DAP of 386.77 µGy·m^2^. The detailed CBCT scanning protocols, including kVp, mA, ms, and DAP, are summarized in Table 1. Representative images from each protocol are shown in Figure 2.

### 2.4. AI Processing

AI-based image post-processing was performed using a pre-trained deep learning model designed for CBCT image enhancement. The model architecture was based on an Attention U-Net-type convolutional neural network for image-to-image post-processing. Model training was completed before the present study using paired low- and high-dose CBCT datasets acquired from skull phantom experiments, and no additional training, fine-tuning, or parameter adjustment was conducted using the CBCT images obtained in this study. For the original model development, the skull phantom dataset was divided into training and validation subsets; however, no further validation or model optimization was performed within the scope of the present study.

During model development, a composite loss function incorporating pixel-wise and perceptual similarity components was used to guide image enhancement. In the present study, the AI model was applied as a fixed post-processing tool to all CBCT datasets to ensure consistent image processing across radiation dose levels. Because this investigation aimed to explore the feasibility of AI-assisted low-dose CBCT imaging rather than to develop or validate an AI model, detailed assessment of model training performance, generalizability, or safety was beyond the scope of this study. Further technical details regarding model development and validation procedures are provided in Supplementary Methods S1.

**Figure 3:**
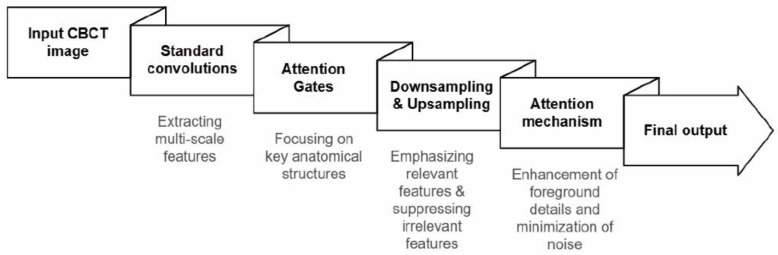
Overview of the Attention U-Net algorithm.

The Attention U-Net architecture incorporates attention-gating mechanisms within skip connections, enabling selective modulation of feature propagation to emphasize salient anatomical structures while suppressing background noise. Multiscale features are extracted through standard convolutional operations and subsequently refined by Attention Gates using contextual information to reconstruct denoised CBCT images (Figure 3).

### 2.5. Subjective Clinical Evaluation

Subjective visual grading was selected as the primary outcome measure to align with the exploratory aim of this feasibility study and to focus on clinician-perceived image quality. Objective image quality metrics, such as peak signal-to-noise ratio (PSNR) and structural similarity index (SSIM), were intentionally excluded as primary outcomes. In an in vivo clinical setting, unlike stationary phantom experiments, subtle patient micro-movements and repositioning between scans lead to inevitable anatomical misregistration. Because pixel-wise similarity metrics are highly sensitive to even sub-millimeter shifts, they may yield mathematically penalized scores that do not reliably correlate with actual diagnostic visibility. Consequently, this study prioritized multi-expert subjective visual grading to assess the perceptibility of critical diagnostic landmarks relevant to routine clinical interpretation. The clinical image quality of the six CBCT image sets was independently evaluated by five experienced clinicians: four specialists in oral and maxillofacial surgery and one specialist in dental radiology. All evaluations were performed using DICOM viewer software (Rainbow 3D Viewer, version 1.1.0; Dentium). Evaluators were permitted to adjust image display parameters, such as density and contrast, to reflect routine clinical viewing conditions.

A total of 12 criteria were assessed, representing key structures relevant to implant planning, orthodontic assessment, and endodontic diagnosis.

(1) sinus floor and cortex in the left maxillary first molar region;
(2) cortex of the alveolar crest in the left maxillary first molar region;
(3) lamina dura and periodontal ligament (PDL) space of the mesiobuccal root of the left maxillary first molar;
(4) trabecular pattern in the left maxillary first molar region;
(5) cortex of the mandibular canal in the right mandibular first molar region;
(6) cortex of the alveolar crest in the right mandibular first molar region;
(7) lamina dura and PDL space of the mesial root of the right mandibular first molar;
(8) trabecular pattern in the right mandibular first molar region;
(9) intermaxillary suture;
(10) overall image quality for orthodontic diagnosis;
(11) overall image quality for periapical lesion diagnosis;
(12) overall image quality for implant treatment planning.

**Figure 4:**
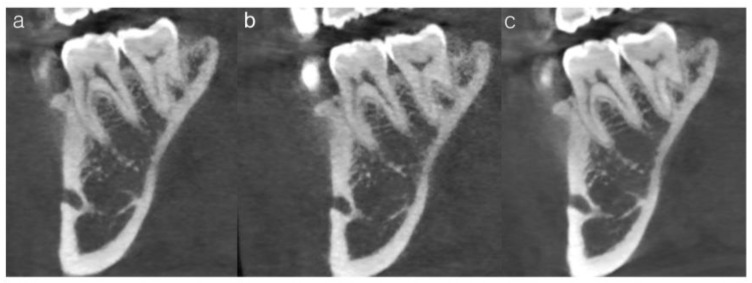
Representative CBCT images illustrating subjective image quality ratings for the trabecular pattern of tooth #46. (**a**) Insufficient image quality (mean score = 3.3), showing poor visualization of the trabecular architecture. (**b**) Acceptable but suboptimal image quality (mean score = 4.0), in which trabecular structures are partially discernible. (**c**) Optimal image quality (mean score = 5.1), demonstrating clear and continuous trabecular patterns.

Each criterion was scored on a six-point scale, with higher scores indicating clearer anatomical visualization. All images were independently evaluated in a randomized order, and the evaluators were blinded to both radiation dose level and AI processing status. Each clinician completed the assessment twice, one month apart, to determine intra-rater reliability. Representative examples of insufficient, acceptable, and optimal image quality based on the subjective scoring system are provided in Figure 4.

### 2.6. Statistical Analysis

To examine differences in overall image quality among the CBCT groups, a repeated-measures analysis of variance (ANOVA) was performed on overall image quality scores, followed by Tukey’s Honestly Significant Difference (HSD) post hoc test for pairwise comparisons. Overall image quality scores were calculated by averaging all scores across the 12 evaluation criteria from the five raters for each CBCT image, thereby summarizing the overall perceived image quality for each imaging condition. This approach was employed to minimize rater-specific and criterion-specific variability and to focus on the overall diagnostic utility of the AI-enhanced images. Inter-rater reliability was estimated using the intraclass correlation coefficient (ICC), whereas intra-rater reliability was assessed using Pearson’s correlation coefficient (*r*) between the two evaluations. A significance threshold of *p* < 0.05 was adopted. All analyses were performed using SPSS Statistics, version 25.0 (IBM Corp., Armonk, NY, USA).

## 3. Results

### Subjective Clinical Efficacy Evaluation

The average clinical evaluations of the CBCT images are summarized in Table 2. Based on subjective assessments, the 100% dose raw image (Image 5) received the highest score, followed by the AI-processed 20% dose image (Image 4) and the 20% dose raw image (Image 3). The lowest score was assigned to the 10% dose raw image (Image 1). The overall ranking of the image quality scores was as follows: Images 5, 4, 3, 6, 2, and 1.

Overall, AI processing improved the clinical evaluation scores of low-dose CBCT images. At the same dose levels, AI-processed images consistently received higher scores than their raw counterparts. Specifically, the AI-processed 20% dose images outperformed the corresponding 20% raw images, and the AI-processed 10% dose images achieved higher scores than the 10% raw images. However, in the 100% dose group, AI-processed images were rated lower than the raw images.

Repeated-measures ANOVA revealed a statistically significant difference between the image groups (*p* < 0.001). Tukey’s HSD post hoc analysis indicated that the AI-processed 10% dose image had significantly lower overall image quality scores than the 100% dose raw image (median 4.25, range 2.90–5.10 vs. median 5.05, range 4.50–5.50; *p* = 0.0074). Although this numerical difference (mean 4.03 vs. 5.11) was statistically significant, it should be interpreted with clinical caution, as the AI-processed 10% dose image maintained a mean score above 4.0, corresponding to a clinically acceptable image quality level. In contrast, the AI-processed 20% dose image showed no significant difference from the 100% dose raw image (median 4.45, range 3.50–5.30 vs. median 5.05, range 4.50–5.50; *p* > 0.05).

Notably, inter-rater reliability, as assessed by the ICC, was low (ICC = 0.280), indicating substantial variability in subjective image quality ratings among evaluators. This limited agreement among evaluators represents a critical finding and should be considered when interpreting differences in overall subjective scores. In contrast, intra-rater reliability, assessed using Pearson’s correlation coefficient, demonstrated moderate agreement (r = 0.735).

## 4. Discussion

The advent of CBCT in the early 2000s revolutionized three-dimensional imaging of the head and neck by providing high image quality together with advantages such as cost-effectiveness and compact design [29,30]. According to the Health Protection Agency (United Kingdom), the radiation dose from a dental CBCT scan is generally lower than that of dental multidetector computed tomography; however, it remains approximately 2–45 times higher than that of conventional two-dimensional panoramic imaging [31]. Therefore, clinicians should conduct comprehensive discussions with patients and obtain informed consent prior to CBCT imaging, ensuring adherence to the ALARA principle to minimize unnecessary radiation exposure [32].

In the present study, radiation dose reduction was achieved using manufacturer-defined low-dose CBCT acquisition protocols that involved coordinated adjustments of tube voltage (kVp), tube current (mA), acquisition sequence, and the number of projections, together with the application of additional filtration. Although these modifications effectively reduced radiation exposure, as reflected by the system-reported dose–area product (DAP), they were associated with increased image noise and degradation of fine anatomical details. The present findings suggest that AI-based post-processing may serve as a supportive tool for mitigating image degradation associated with radiation dose reduction, particularly within a moderate low-dose range. This consideration is especially relevant for pediatric patients, who carry a higher lifetime risk of radiation-induced harm than adults for an equivalent radiation dose [6,33]. Given the expanding use of dental CBCT in implantology, periodontology, and orthodontics [3,34,35], careful task-specific implementation of AI-assisted dose reduction strategies is warranted.

Although statistically significant differences were observed at the lowest dose level, the absolute differences in image quality scores were relatively small, indicating that statistical significance does not necessarily correspond to a meaningful loss of clinical relevance. For the 10% dose images, AI processing improved overall image appearance; however, residual noise and reduced structural clarity may still limit diagnostic confidence for tasks requiring precise visualization of fine anatomical details.

In this study, inter-rater reliability was relatively low (ICC = 0.280), indicating limited agreement among evaluators. This variability may be attributed to the subjective nature of interpreting subtle anatomical structures under varying noise levels in low-dose CBCT images. In addition, AI-based post-processing can modify image texture and contrast characteristics through noise suppression, which may alter visual cues used for image interpretation. Such changes may introduce evaluator-dependent perceptual bias, as some clinicians may prefer the familiar graininess of raw low-dose images for edge delineation, whereas others may favor the visually cleaner appearance of AI-processed images.

Furthermore, the observed discrepancies in image quality scores suggest that the perceived performance of the AI model may differ according to the evaluator’s clinical focus. For example, evaluators emphasizing bone density or fine trabecular detail may regard AI-processed images as less reliable due to contrast-dependent smoothing, whereas those focusing on overall morphological assessment may assign higher scores. Collectively, these factors—including inherent limitations of the AI model and evaluator-dependent preferences—likely contributed to increased inter-observer variability.

Unexpectedly, the AI-processed 100% dose images did not outperform the 100% raw dose images and, for some evaluation criteria, were rated lower than the 20% raw images. One possible explanation is that applying AI-based enhancement to images that already exhibit high intrinsic quality may introduce over-smoothing or subtle alterations in image contrast, thereby obscuring fine anatomical details and reducing perceived diagnostic clarity. In addition, evaluator-related perceptual bias may have contributed to this outcome. Clinicians are generally accustomed to the texture and noise characteristics of conventional high-dose CBCT images, whereas AI-induced changes in image appearance may be perceived as artificial or less reliable. Furthermore, the AI model used in this study was primarily optimized to compensate for noise-related degradation in low-dose images, and its application to standard-dose images may exceed the intended operating range of the model, thereby limiting its effectiveness. Collectively, these observations suggest that caution is warranted against the indiscriminate application of AI post-processing to already high-quality images and emphasize the need for dose- and task-specific validation of AI-based image enhancement techniques.

In recent years, a growing body of research has actively explored the application of artificial intelligence to CBCT imaging across oral and maxillofacial disciplines, aiming to overcome inherent limitations related to image noise, artifacts, and reduced acquisition protocols [36]. These studies reflect diverse clinical objectives, including image enhancement, anatomical segmentation, and task-specific diagnostic support. For example, deep learning–based approaches have been proposed for robust mandible segmentation in dental CBCT scans affected by metal artifacts, demonstrating the potential of AI to improve anatomical delineation under challenging imaging conditions [17]. In addition, AI-based post-processing of fast-scan low-dose CBCT has been shown to enhance image quality for head and neck adaptive radiotherapy, highlighting the broader applicability of AI-enhanced CBCT beyond conventional dental diagnostics [18].

With respect to maxillary sinus evaluation, previous studies have reported that AI-enhanced CBCT imaging can improve diagnostic discrimination between pathological and normal conditions. Kim et al. [26] demonstrated that internally denoised, AI-enhanced CBCT images enabled reliable differentiation among fungal ball, sinusitis, and normal maxillary sinus cases, indicating the potential utility of AI-based image processing for sinus pathology assessment. However, the present findings indicate that the visibility of thin cortical structures, such as the maxillary sinus floor, remains suboptimal under low-dose conditions despite AI-based post-processing. This limitation is clinically relevant given the widespread reliance on CBCT to prevent sinus floor perforation during implant placement [37], and it suggests that the effectiveness of AI enhancement may be constrained by anatomical thickness and contrast characteristics in specific regions.

In contrast, the mandibular canal remained relatively well visualized even in AI-processed low-dose CBCT images. Accurate identification of the inferior alveolar nerve (IAN) is essential for avoiding nerve injury during implant surgery, and AI-based image enhancement may help preserve the visibility of such critical diagnostic structures under substantially reduced radiation exposure. Recent studies using generative AI-enhanced low-dose CBCT have similarly reported significant improvements in the visibility of the mandibular canal and periodontal ligament in the evaluation of impacted mandibular third molars [27]. These observations are consistent with the present findings regarding the relative robustness of mandibular canal visualization. Collectively, these results suggest that the performance and clinical utility of AI-enhanced CBCT depend on both anatomical region and diagnostic task, underscoring the importance of region-specific evaluation in low-dose imaging contexts.

Although repeated CBCT imaging in a healthy subject raises ethical concerns, this intra-individual design was an intentional methodological choice aimed at minimizing biological noise, such as inter-individual anatomical variations that can mask subtle technical effects of AI processing. By isolating radiation dose and AI-based enhancement within a strictly controlled model, this study established a high-fidelity technical baseline for feasibility assessment. Nevertheless, the use of a single healthy adult male without dentomaxillofacial pathology limits the generalizability of the findings and precludes the application of task-based diagnostic performance metrics, such as receiver operating characteristic (ROC) analysis. Accordingly, the present results should be interpreted strictly within the context of technical feasibility rather than clinical effectiveness.

Furthermore, while quantitative image quality indices such as the structural similarity index (SSIM) are widely used in phantom-based AI research, their applicability to clinical feasibility studies involving live subjects remains limited. In in vivo imaging, the technical challenge of achieving precise three-dimensional registration between repeated scans means that pixel-wise similarity metrics may underestimate perceived diagnostic image quality due to minor anatomical misregistration and unavoidable patient motion. Therefore, this study prioritized expert-led qualitative evaluation to better reflect real-world clinical image interpretation. Nevertheless, future investigations conducted under carefully controlled conditions, such as phantom-based or ex vivo studies, may incorporate objective image quality metrics to enable direct technical comparison with existing literature.

This study was also conducted under relatively ideal imaging conditions, as no metallic restorations, such as crowns or dental implants, were present. In routine clinical practice, artifacts caused by metallic restorations and implants are common and may affect the performance of AI-based image processing. Therefore, additional validation under these conditions is needed. Furthermore, the evaluation criteria in this study focused mainly on a limited number of anatomical landmarks in the first molar region. Given the expanding use of CBCT in daily dental practice, future studies should include additional clinically important structures, such as the mental foramen in the second premolar region. However, in the present study, the evaluation scope was intentionally limited to maintain consistency and to reduce interobserver variability. Finally, although image evaluation was performed using a clinical-grade monitor rather than a diagnostic radiology display, this setup reflects typical real-world clinical viewing conditions.

Accordingly, the present findings should be interpreted strictly as preliminary technical feasibility observations rather than as evidence of clinical effectiveness. The conclusions are framed within the context of whether AI-based post-processing can maintain the visibility of critical anatomical landmarks under reduced dose protocols, rather than establishing definitive diagnostic superiority. Despite these limitations, this study provides valuable insight into the potential of AI-based image enhancement for low-dose CBCT imaging. Future research including a wider range of patient ages, sexes, anatomical variations, and pathological conditions will be important to more comprehensively evaluate AI performance in routine clinical settings. Ultimately, a randomized controlled trial comparing low-dose and standard-dose CBCT in patients undergoing oral and maxillofacial surgical procedures, such as implant placement or extraction of impacted third molars, would help clarify the clinical value of AI-assisted low-dose CBCT imaging.

## 5. Conclusions

This pilot study demonstrates the feasibility of AI-based image processing for improving image quality in low-dose CBCT imaging. Under controlled conditions, AI-processed 20% dose images exhibited image quality comparable to that of standard-dose CBCT based on subjective visual assessment, supporting the technical feasibility of radiation dose reduction. Given the exploratory single-subject design, these findings should be interpreted with caution. Expanded and standardized evaluation criteria and large-scale studies are essential for integrating AI-enhanced CBCT into clinical practice.

## Figures and Tables

**Table 1:** The detailed CBCT scanning parameters for the six image sets.

Image	Dosage	DAP (µGy·m^2^)	AI Process	kVp	mA	Exposure Time (ms/Projection)	Projections	FOV (cm)
1	10%	193.38		85	8.5	16	480	12 × 9.5
2	10%	193.38	AI	85	8.5	16	480	12 × 9.5
3	20%	386.77		85	9	16	750	12 × 9.5
4	20%	386.77	AI	85	9	16	750	12 × 9.5
5	100%	1933.83		95	11	12	400	12 × 9.5
6	100%	1933.83	AI	95	11	12	400	12 × 9.5

**Table 2:** Subjective image quality evaluation of CBCT images under varying dose and AI conditions.

Image No.	1	2 (AI)	3	4 (AI)	5	6 (AI)
Sinus floor cortex of #26	4.4	4.2	4.6	4.5	5.5	4.7
Cortex of alveolar crest of #26	3.9	4.6	4.4	4.2	5.5	4.7
Lamina dura, PDL space of #26	3.1	3.2	3.8	3.5	4.5	3.9
Trabecular pattern of #26	3.2	2.9	4.1	4.4	4.9	3
Cortex of mandibular canal of #46	4.3	4.7	4.4	4.6	5	4.8
Cortex of alveolar crest of #46	4.1	4.3	4.8	4.2	5	5
Lamina dura, PDL space of #46	3.1	3.4	3.7	3.6	4.5	4.2
Trabecular pattern of #46	3.3	3.3	4	4.9	5.1	3.8
Intermaxillary suture	4.3	4.5	4.7	4.6	5.3	4.6
Overall image quality of orthodontic diagnosis	4.7	4.6	5.2	5.3	5.5	4.9
Overall image quality for periapical lesion	4.1	3.6	3.7	4.3	5.1	2.7
Overall image quality for implant planning	5	5.1	5.1	5.2	5.4	5
Mean ± SD	3.96 ± 0.37 *	4.03 ± 0.63 *	4.38 ± 0.53	4.44 ± 0.50	5.11 ± 0.25	4.28 ± 0.14 *

* indicates a statistically significant difference (*p* < 0.05) compared with Image 5, as determined by repeated-measures ANOVA followed by Tukey’s HSD post hoc test. Here, #26 and #46 refer to tooth numbers based on the FDI two-digit notation system, where #26 represents the maxillary left first molar and #46 represents the mandibular right first molar.

## Data Availability

The data presented in this study are available on request from the corresponding author due to ethical restrictions regarding patient confidentiality.

## Supplementary Materials

The following supporting information can be downloaded at: https://www.mdpi.com/article/10.3390/bioengineering13030304/s1. Supplementary Methods S1. Detailed description of the AI model development process and transparency statement. Supplementary Table S1. Complete raw dataset including all individual inter-rater scores and intra-rater duplicate evaluations.

## Abbreviations

AI	Artificial intelligence
ALARA	As low as reasonably achievable
ANOVA	Analysis of variance
CBCT	Cone-beam computed tomography
CNN	Convolutional neural networks
DAP	Dose–area product
FOV	Field of view
HSD	Honestly significant difference
IAN	Inferior alveolar nerve
ICC	Intraclass correlation coefficient
KHIDI	Korea Health Industry Development Institute
NIPA	National IT Industry Promotion Agency
