# Supplementary material for: Feasibility of Artificial Intelligence-Processed Low-Dose Cone-Beam Computed Tomography in Dental Imaging
Source: Bioengineering (Basel). 2026 Mar 5;13(3):304. doi: 10.3390/bioengineering13030304 (PMC13024084; doi:10.3390/bioengineering13030304)
Supplement: Supplementary file 1 [file bioengineering-13-00304-s001.zip › Supplementary_Methods_S1.pdf]

Supplementary Methods S1. AI Model Development and Transparency Statement.

S1.1. Model Architecture

The AI-based image enhancement model used in this study was implemented using an Attention U-Net-type convolutional neural network architecture, originally described by Oktay et al. (2018). The network follows an encoder–decoder framework with skip connections, incorporating attention-gating mechanisms to selectively modulate feature propagation. These attention gates enable suppression of irrelevant background activations while emphasizing diagnostically relevant anatomical structures.

The model was designed for image-to-image post-processing of reconstructed CBCT volumes, with the objective of noise reduction and contrast enhancement under reduced radiation dose conditions.

S1.2. Training Dataset

Model training was completed prior to the present investigation. Approximately 4,000 paired low-dose and corresponding high-dose CBCT slices were used during development. All datasets were acquired using a standardized skull phantom model to eliminate biological variability and ensure controlled acquisition conditions.

The phantom dataset was divided into training and validation subsets during model development. No CBCT images obtained in the present clinical feasibility study were used for training, fine-tuning, or parameter optimization.

S1.3. Preprocessing Procedures

Prior to training, the following preprocessing steps were applied:

- Spatial resolution normalization to ensure dimensional consistency within the encoder–decoder architecture and to prevent mismatch during up- and down-sampling operations.
- Exposure to multiple spatial resolutions during training to improve robustness across different scan modes.
- Intensity normalization to 32-bit floating-point format, scaled to the 0–1 range, to ensure numerical stability during loss computation.

S1.4. Loss Function Composition

During model development, a composite loss function was employed to balance structural fidelity and perceptual similarity. The loss function consisted of:

- L2 (mean squared error) loss,
- Perceptual loss based on feature maps extracted from a pretrained VGG-19 network,
- Structural Similarity Index Measure (SSIM) loss.

The relative weighting of these components was optimized empirically to minimize noise amplification while preserving fine anatomical structures.

The Attention U-Net backbone was trained from scratch using CBCT data. The pretrained VGG-19 network was utilized solely for perceptual feature extraction in loss computation and was not fine-tuned on CBCT data.

S1.5. Overfitting Control and Optimization Strategy

To mitigate overfitting during development, the following strategies were implemented:

- Data augmentation, including rotation, flipping, and inversion,
- Learning rate scheduling using a StepLR decay strategy,
- Continuous monitoring of validation loss with checkpoint-based selection of the optimal training epoch.

S1.6. Validation and Safety Considerations

Training and validation were performed using phantom-acquired datasets. During model development, independent internal clinical CBCT datasets (not used in training) were qualitatively assessed to monitor generalizability and potential artifact generation.

Model outputs were reviewed to minimize the risk of artificial structure creation or unintended anatomical distortion. A staged field validation approach was implemented during development, whereby model outputs were evaluated in controlled clinical environments prior to final stabilization.

S1.7. Application in the Present Study

In the present investigation, the AI model was applied strictly as a fixed post-processing tool. No retraining, fine-tuning, or parameter adjustment was performed using the study data.

This study aimed to evaluate the clinical feasibility of AI-assisted low-dose CBCT imaging rather than to validate the AI model itself. The model was used under fixed inference settings to ensure consistent processing across radiation dose levels.
